# Healthy Foods, Healthy Families: combining incentives and exposure interventions at urban farmers’ markets to improve nutrition among recipients of US federal food assistance

**DOI:** 10.15171/hpp.2016.02

**Published:** 2016-03-31

**Authors:** April B. Bowling, Mikayla Moretti, Kayla Ringelheim, Alvin Tran, Kirsten Davison

**Affiliations:** ^1^Department of Nutrition, Harvard School of Public Health, 665 Huntington Avenue, Boston, MA 02115, USA; ^2^Farm Fresh Rhode Island, 1005 Main Street, Pawtucket, RI 02860, USA

**Keywords:** Obesity, Socioeconomic factor, Diet quality, Farmers markets, Food assistance, Family nutrition intervention

## Abstract

**Background:** Healthy Foods, Healthy Families (HFHF) is a fruit and vegetable (F&V) exposure/incentive program implemented at farmers’ markets in low-income neighborhoods, targeting families receiving US federal food assistance. We examined program effects on participants’ diet and associations between attendance, demographics and dietary change.

**Methods:** Exposure activities included F&V tastings and cooking demonstrations. Incentives included 40% F&V bonus for electronic benefit transfer (EBT) card users and $20 for use purchasing F&V at every third market visit. Self-report surveys measuring nutritional behaviors/literacy were administered to participants upon enrollment (n = 425, 46.2% Hispanic, 94.8%female). Participants were sampled for follow-up at markets during mid-season (n = 186) and at season end (n = 146). Attendance was tracked over 16 weeks.

**Results:** Participants post-intervention reported significantly higher vegetable consumption(P = 0.005) and lower soda consumption (P = 0.005). Participants reporting largest F&V increases attended the market 6-8 times and received $40 in incentives. No change in food assistance spent on F&V (P = 0.94); 70% reported significant increases in family consumption of F&V,indicating subsidies increased overall F&V purchasing. Participants reported exposure activities and incentives similarly affected program attendance.

**Conclusion:** Interventions combining exposure activities and modest financial incentives at farmers’ markets in low-income neighborhoods show strong potential to improve diet quality of families receiving federal food assistance.

## Introduction

Obesity is a major public health concern in the United States, disproportionately affecting low-income and minority families and producing long-term chronic disease consequences.^1^ Diet quality is an important component of weight regulation and positive health.^2,3^ High rates of obesity among low-income individuals are likely due, in part, to lower diet quality observed in this vulnerable population,^4^ which may reflect the limited access and exposure to fruits and vegetables (F&Vs) observed in urban settings in developed countries throughout the world.^5^ Indeed, poor eating patterns among low-income and inner-city populations are strongly associated with lack of exposure to F&V and high allostatic loads, which together may drive a preference for high-satiety processed foods and sugar-sweetened beverages. Urban farmers’ markets, when developed in low-income and minority neighborhoods, are one avenue to improve access and exposure to F&V^6^ and potentially reduce the risk of obesity in this population.

Several matched and bonus monetary incentive programs for food assistance recipients in the United States have been implemented to encourage the purchase of fresh F&V at farmers’ markets in under-served communities.^7-9^ Coupled with expanded acceptance of electronic benefit transfer (EBT) cards at markets, incentive programs in cities such as New York, Philadelphia, and San Diego have proven effective at increasing the amount of food assistance dollars spent at farmers’ markets, as well as improving self-reported fresh F&V consumption among participants.^8,9^

While these programs may successfully improve access to F&V among target populations adjacent to farmers’ markets,^10^ they do not directly address issues of exposure to and acceptance of F&Vs. Recent studies of farmers’ market use among recipients of the US Federal Supplemental Nutrition Assistance Program (SNAP) indicate that barriers to exposure – which includes availability in the home, cooking, serving, and tasting of F&V – may be as significant as access barriers in limiting F&V consumption and increasing intake of nutrient-poor, calorie-dense foods.^11,12^ That is, parents may decline to purchase F&V because they cannot expend their limited budget on foods that will go to waste.^10^

Exposure interventions are defined here as activities which encourage tasting and acceptance of F&V, and include F&V tastings, cooking demonstrations, peer F&V eating environments, and family nutritional education. Access/incentive interventions include placement of markets in underserved neighborhoods and provision of financial subsidies, and directly improve the ability of families to obtain F&V. Therefore, access interventions are complemented by exposure interventions, which encourage consumption of increased F&V quantities and diversity after access is obtained. Furthermore, there is substantial evidence that conducting exposure interventions that enable children to taste F&V in novel environments may improve child acceptance, and therefore, increased consumption of F&V.^13^

Farm Fresh Rhode Island (FFRI), a non-governmental organization with the mission of providing access to local food in Rhode Island, has begun implementing a program that seeks to addresses not only F&V access, but also exposure and acceptance among federal food assistance recipients. Healthy Foods, Healthy Families (HFHF) is implemented in six farmers’ markets in low-income, urban neighborhoods from July through October each year. Families with at least one child under the age of 12, and who participate in at least one of the US food assistance programs Women, Infants and Children (WIC) or SNAP, may enroll to receive financial incentives to purchase F&V in exchange for participating in family-based nutrition exposure and education interventions held weekly at farmers’ markets.

This study was conceived as a feasibility and efficacy analysis using existing process evaluation data collected by the HFHF program during the summer of 2013. Our specific research aims included the following: (1) analyze and disseminate programmatic data on financial benefits and exposure activities; (2) examine effects of HFHF participation on participants’ F&V and soda consumption; (3) investigate the program’s effect on WIC/SNAP budget F&V expenditure patterns and use of food assistance at participating farmers’ markets; and (4) explore the relative importance of financial (access) incentives and exposure interventions as drivers of participant enrollment and retention, as well as participants’ perceptions of barriers, support, and benefits from HFHF participation.

## Materials and Methods

### Ethics of human subject participation

This research protocol was reviewed and exempted by the Harvard T.H. Chan School of Public Health Institutional Review Board.

### Setting 

FFRI began the HFHF program in 2008 with assistance from the University of Rhode Island, aiming to empower low-income Rhode Island families to shop for and cook more affordable fresh foods by providing access, education and financial incentives at urban farmers’ markets. The program is implemented at six farmers’ markets in Providence over a 17-week season.

### Design 

HFHF is composed of exposure interventions, such as tasting opportunities and educational activities, and access interventions, such as F&V subsidies, and participation incentives. These interventions are detailed in Table 1. SNAP recipients receive a 40% matched monetary incentive when they use their EBT card at these markets, regardless of HFHF participation. However, at every third market they attend, HFHF participants also receive $20 in “Bonus Buck” tokens, up to $120. These tokens could only be used to purchase F&V at the farmers’ market. Exposure activities varied by week, with cooking demonstrations held on a more limited basis, while tasting opportunities, recipe cards, and children’s educational activities were available every week. All activities were conducted in English or Spanish, materials were available in both languages, and staff included bilingual and bicultural educators. Participants were allowed to attend any participating market, and could attend multiple markets per week.

**Table 1:** HFHF exposure interventions and incentives

**Exposure interventions**	**Incentives**
Monthly: Healthy cooking demonstrations	First attendance: Children's Book: The Best Me/El Mejor Yo
Weekly: Children’s nutritional literacy activities and taste tests	Second attendance: Reusable Canvas Shopping Bag
Weekly: Recipe cards and adult education materials	Third attendance: $20 in Bonus Bucks for purchase of fresh fruits & vegetables at farmers’ markets
	Every third Market Attended Thereafter: additional $20 Bonus Bucks (limited to $120 per family per season)
	Weekly: 40% EBT card bonus ($2 in Bonus Bucks for each $5 of EBT card spending at the market)

Abbreviation: HFHF, Healthy Foods, Healthy Families; EBT, electronic benefit transfer.

### Participants

Any Rhode Island family enrolled in WIC or SNAP with at least one child under the age of 12 was eligible to participate in the HFHF program. Enrollment occurred at a sign-up table set up weekly throughout the season at each of the participating farmers’ markets. For one month prior to the July start date, HFHF was advertised through flyers at local health clinics, community centers, neighborhoods, and through direct referrals from WIC offices. Enrollment and programs were conducted in both English and Spanish. In order to enroll, one adult family member completed a sign-up sheet and a pre-program survey on nutritional behaviors and nutritional literacy.

Enrollment was continued until the maximum number of participants (400) supported by funding were enrolled, at which time a waiting list was started. Enrollees who did not attend another market within the first 4 weeks of the program were contacted and asked if they intended to continue in the program. Those who indicated that they did not wish to continue, or who said they did wish to continue but did not subsequently attend a market within 2 weeks were withdrawn and replaced by a family on the waiting list. Twenty-five wait-listed families were eventually moved into the program to replace families who withdrew. Participants were instructed to check-in at the HFHF table located at each market upon their arrival in any given week, where participant attendance and Bonus Bucks received were tracked by ID number, and surveys could be administered when appropriate.

### Evaluation

The HFHF program was evaluated using a pre-post survey design. Self-report surveys measuring nutritional behaviors and literacy were administered by HFHF staff and translators to all HFHF (n=425) enrollees upon entry into the programs, after they provided consent. The programmatic data collection did not include a control group. Due to limited resources it was not feasible to actively track participants over time and ensure follow-up for all enrollees at mid-season and end of season. Thus, Farm Fresh Rhode Island elected to sample enrollees from each farmers’ market site at specified time points. Mid-season surveys of program satisfaction and barriers to attendance were administered to HFHF attendees in August, with 2 days of survey administration at each market during the month. Participants received and completed a mid-season survey if they attended a specific market on the day of assessment (n=186). Likewise, exit surveys of nutritional behaviors and literacy were administered to HFHF enrollees during the last 4 weeks of the program. Participants received and completed an exit survey if they attended the market being surveyed that day (n=146).

### Measures

A summary of the types of data collected and respective survey instruments is contained in Table 2.

**Table 2:** Constructs and evaluation instruments

**Construct**	**Instrument**
Attendance	Recorded at check-in table at all markets
Benefits received	Recorded and distributed at check-in table at all markets
Participation in exposure activities	Staff activities report (qualitative)
Exposure outcomes	Pre-program and post-program survey questions:
	Has your family tried any new F&Vs at HFHF that you had not eaten before?
Food assistance expenditure patterns	Pre-program and post-program survey questions:
	How much of your family’s weekly WIC/SNAP budget is spent on F&Vs?
F&V and soda intake	Pre-program and post-program survey questions:
	How many times do you consume soda on a daily basis?
	On an average day, how many times do you have a vegetable to eat?
	On an average day, how many times do you have a fruit to eat?
	Does your family eat more fresh F&Vs as a result of participating in HFHF?

Abbreviations: HFHF, Healthy Foods, Healthy Families; F&V, fruits and vegetables; SNAP, Supplemental Nutrition Assistance Program; WIC, Women, Infants and Children Nutritional Assistance Program.

*Demographics*

Demographic information including age, number and ages of children, zip code, ethnicity, preferred language, insurance status, and food assistance enrollment, was obtained through the enrollment form.

*Outcomes*

The pre- and post-program surveys assessed the enrolled parents’ participation objectives, F&V intake, soda intake, food assistance spending patterns, and barriers to participation. The surveys did not ask parents to assess their children’s diets. The mid-season survey assessed program satisfaction, efficacy, and barriers to attendance. The number of questions ranged from 6 on the mid-season survey, to 17 on the exit survey, to 27 on the entrance survey, including demographic questions.

The program was evaluated using internally designed surveys that were worded to provide comparability with major indicator studies where practically feasible. For example, for comparison to the *2013 CDC State Indicator Report on Fruits and Vegetables*,^14^ surveys asked “times per day” instead of “servings per day” for soda, vegetable, and fruit consumption.

*Process measures*

Attendance, markets frequented, Bonus Bucks received, and surveys completed were tracked by program ID number using a participant check-in system at each market for the duration of the season. This was facilitated by requiring participants to check-in upon arrival in order to receive attendance credit towards their next Bonus Bucks installment. Attendance acted as a proxy for participation in exposure activities, which was not explicitly tracked; qualitative assessments by staff indicated that most attendees participated in exposure activities at least passively. Participants were questioned regarding relative importance of financial incentives and exposure activities to their attendance on exit surveys as well.

*Analysis*

Descriptive statistics were used to summarize participant demographics and process measures such as attendance, benefits received and WIC/SNAP expenditures at markets. Given the non-normal distributions of dietary change data, paired Wilcoxon signed-rank tests were used to assess pre- and post-intervention differences in fruit, vegetable, and soda consumption. Multiple regression analysis was used to assess associations between demographics, attendance, total Bonus Bucks received, and dietary changes. Rather than calculating total dietary change scores, changes in vegetable consumption, fruit consumption, and soda consumption were treated as separate, continuous dependent variables as the more principled approach. Demographic variables were added in a step-wise fashion and retained only if significant at α=0.1. Relationships between dietary change outcomes and independent variables of attendance and Bonus Bucks received were assessed controlling for significant demographic variables.

## Results

### Demographics, retention and program utilization

Table 3 illustrates the demographics of the participants served by the 2013 program and participants captured during follow-up survey sampling. Of the 425 families initially enrolled, 94.8% were signed-up by an individual who self-identified as a female guardian, 46.2% identified as Hispanic, and 74.6% were enrolled in SNAP. Entry surveys were completed for all 425 families, mid-season surveys conducted at all the markets were completed by 186 families, and exit surveys conducted at all the markets were completed by 146 families. Heterogeneity tests showed no significant demographic differences between those completing exit surveys and the general HFHF participant pool, however, those who withdrew from the program were a lower percentage self-identified Asian (*P*=0.03), a higher percentage self-identified multi-ethnic (*P*=0.003), and more likely to spend little to none of their WIC/SNAP budget on fruits and vegetables (*P*=0.04).

**Table 3:** HFHF characteristics by survey sample

**Measure**	**Baseline**	**Mid-Season**	**Exit**
Total participants in 2013, N	425	186	146
Mean age of respondent (range)	34.5 (17-68)	35.5 (19-64)	35.6 (20-64)
Female respondents, N (%)	401 (94.8)	176 (94.6)	140 (95.9)
Mean age of child (SD)	5.7 (3.2)	5.7 (3.2)	5.7 (3.2)
Mean number of children (SD)	2.1 (1.1)	2.2 (1.0)	2.1 (1.0)
Mean number of adults in household (SD)	2.2 (1.1)	2.3 (1.0)	2.3 (1.1)
Race/ethnicity, N (%)			
Asian	37 (8.9)	30 (16.1)	23 (15.8)
Black	33 (7.9)	13 (6.9)	11 (7.5)
Cape Verdean	3 (0.7)	1 (0.5)	1 (0.7)
Hispanic	193 (46.2)	86 (46.2)	65 (44.5)
Multi-Ethnic	20 (4.8)	4 (2.7)	3 (2.1)
White	126 (30.1)	47 (25.3)	39 (26.7)
Other/unknown	13 (3.1)	3 (1.6)	4 (2.7)
Preferred language, N (%)			
English	233 (55.2)	83 (44.9)	68 (46.6)
Spanish	159 (37.7)	76 (41.1)	57 (39.0)
Other	30 (7.1)	26 (4.0)	21 (14.4)
Health insurance type, N (%)			
Neighborhood/RiteCare	332 (79.1)	149 (80.5)	116 (79.5)
Private	42 (10.0)	17 (9.2)	14 (9.6)
Medicare	25 (5.9)	11 (6.0)	9 (6.2)
Uninsured/unknown	26 (6.0)	8 (4.3)	7 (4.8)
Receipt of federal food assistance,^a^ N (%)			
WIC	307 (72.2)	138 (74.2)	110 (75.3)
SNAP	317 (74.6)	136 (73.1)	108 (73.9)

Abbreviations: HFHF, Healthy Foods, Healthy Families; SNAP, Supplemental Nutrition Assistance Program; WIC, Women, Infants and Children Nutritional Assistance Program. ^a^All participants were enrolled in WIC, SNAP, or both.

Among all families who remained enrolled (n=359), the average number of visits over 17 weeks was 7.7 (±6.0) per family. The average attendance was 9.7 (±6.3) visits among the exit survey group versus 7.7 among all participants who remained enrolled, which was not statistically significantly different.

A total of 270 participants completed at least 3 visits to a market, earning a financial incentive towards F&V purchase. Among participants who earned an incentive, the average Bonus Bucks received totaled $61.19 (±$34.81). The program distributed a total of $16520 Bonus Bucks to participants supplementing their existing WIC and SNAP budgets with funds exclusively for purchasing F&V at farmers’ markets.^15^ During the summer 2013, Farm Fresh Rhode Island processed more than $61000 in SNAP benefits at its markets, up from $4600 in the previous year.

Attendance (total visits) was strongly positively associated with participants’ self-identification as “Asian” (*P*<0.001), but no other demographic variables. Total financial benefits received are determined by attendance since participants received $20 in Bonus Bucks for use in buying F&V at every third attendance. Total benefits received were positively associated with number of children (*P*=0.04) and Asian ethnicity (*P*=0.04), but not with any other demographic characteristics.

### Pre-post dietary changes

Among participants who completed an exit survey, mean self-reported pre-program consumption levels (times/day) were 0.57 for soda (±0.13), 2.42 for vegetables (±0.19), and 2.71 for fruit (±0.21). Mean self-reported post-program consumption levels (times/day) were 0.43 for soda (±0.12), 2.70 for vegetables (±0.21), and 2.92 for fruit (±0.21). Results of the Wilcoxon signed-rank analysis are presented in Table 4. Overall, significant increases in the daily intake frequency of consuming vegetables (11.6%± 4.7%, *P*=0.005) and significant decreases in the daily frequency of consuming soda (-24.6%±10.5%, *P*=0.005) were observed. While fruit consumption also increased, this difference was only marginally significant (*P*=0.10).

**Table 4:** Paired Wilcoxon signed-rank analysis of fruit, vegetable, and soda consumption at program entrance and exit

**Survey question**	**N**	**Baseline mean (95% CI)**	**Exit mean (95% CI)**	**Difference (%)**	***P*** ** value**
How many times do you consume soda on a daily basis?	146	0.57 (0.44, 0.69)	0.43 (0.31, 0.55)	-0.14 (-24.6)	0.005
On an average day, how many times do you have a vegetable to eat?	146	2.42 (2.23, 2.61)	2.70 (2.48, 2.91)	0.28 (11.6)	0.005
On an average day, how many times do you have a fruit to eat?	146	2.71 (2.50, 2.92)	2.92 (2.71, 3.13)	0.21 (7.7)	0.097

The highest average vegetable consumption change occurred among those who made between 6 and 8 market visits, earning $40 in Bonus Bucks. Multiple regression results revealed that among those participants completing exit questionnaires, there was not a statistically significant association between attendance and changes in F&V or soda consumption. This finding was not surprising given the small sample size and limited between-person variation in F&V consumption change.

### Effects on WIC/SNAP F**&**V budgeting

HFHF participants reported no change in the amount of their WIC/SNAP budget spent on F&V (*P*=0.94). Despite this, on exit surveys 70% of participants reported that program participation had significantly increased their families’ consumption of F&V.

### Relative importance of programmatic incentives and exposure components

As shown in Table 5, participants reported that financial incentives (23.1%) and exposure interventions (21.9%) were equal drivers of their retention, with the fact that their family was eating more F&V as a result of the program being the driver cited by the most participants (34.4%). Table 5 also shows additional mid-season and exit survey results regarding participants’ perceptions of barriers, support, and benefits from HFHF participation.

**Table 5:** Supplemental findings from entrance, mid-season and exit surveys

**Survey question**	**Percent**
Participant motivations: Why do you want to participate in HFHF?^a*^	
Family health	32.7
Nutritional education	31.8
Financial benefit	20.3
Weight of a family member	15.2
Why do you return to HFHF?^b*^	
Family eating more F&V	34.4
Financial incentives	23.1
Children’s activities	21.9
Already at the farmer’s market anyway	20.6
Perceived benefits: Have you noticed a benefit to your family by participating in HFHF?^c^	
Kids eating more fruits and vegetables	81.6
Quality time spent together with family	7.0
I have not noticed a benefit	5.3
Parents/guardians eating more fruits and vegetables	4.4
Feel more connected with community	0.9
Noticed other benefit	0.9
How has HFHF affected where you buy food?^b^	
I shop a lot more at the farmers market than I did before.	76.8
I shop a little more at the farmers market than I did before.	18.3
It has not changed where I buy my food.	4.9
Has your family tried any new fruits and vegetables at HFHF that you had not eaten before?^b^
Yes, at the market.	68.5
No, we did not try any new fruits and vegetables this season.	22.3
Yes, at home.	9.2
Please rate the quality of the HFHF program.^b^	
Excellent	85.3
Good	9.1
Acceptable	4.9
Poor	0.7
Perceived barriers: Is there anything inconvenient about coming to the farmers market?^c^	
No	72.5
Price of food	14.6
Hours of market	5.6
Other	3.3
Transportation	2.8
Language barrier	1.1
Is there another parent/guardian in your family supporting your participation?^c^	
Yes	72.1
No	27.9

Abbreviation: HFHF, Healthy Foods, Healthy Families. *Percentages do not add to 100% because respondents could select multiple items; ^a^ Entrance survey item (n=425); ^b^ Exit survey item (n=146); ^c^ Mid-season survey item (n=186).

## Discussion

The retention and attendance results illustrate high participant engagement in the program, despite modest financial incentives. The significant increases in vegetable consumption found in this study agree with and expand upon previous findings.^8^ Non-significant increases in fruit consumption may be partially explained by the high reported consumption of fruit at entrance to the program, as well as the fact that vegetables dominate farmers’ markets offerings. The small, but statistically significant decrease in soda consumption occurred despite none of the programmatic education efforts being targeted specifically at lowering sugar-sweetened beverage intake. It was not feasible to conduct more reliable measures of dietary intake, such as 24-hour recalls for food frequency questionnaires (FFQs) with participants, however, this preliminary finding of a decrease in soda consumption should be explored further in future studies. Previous studies examining increased F&V consumption have found mixed evidence of commensurate decreases in sugar-sweetened beverages.^16^ We could not find any prior research where soda intake decreased when only F&V intake was targeted.

Importantly, the study findings suggest that participants are using the program’s financial incentives to supplement, rather than replace, their WIC/SNAP F&V budget. Specifically, HFHF participants reported no change in the amount of their WIC/SNAP budget spent on F&V despite 70% of participants reporting that program participation had significantly increased their families’ consumption of F&V. This finding rebukes the argument made by some critics that F&V subsidies provided by programs such as HFHF allow participants to spend a larger percentage of their WIC/SNAP budget on less healthy items. Instead, it appears that the financial incentives offered by the HFHF program, like other monetary incentive programs at farmers’ markets, are being used effectively by participants to increase F&V consumption. The survey data also support the relative importance of HFHF’s exposure and educational components in motivating participants to both enroll and continue to attend the markets. The increase in SNAP expenditures with Bonus Bucks implementation is in keeping with findings at other farmers’ markets implementing similar financial incentives for SNAP users.

A third of participants cited the fact that their families were eating more F&V as a result of the program as the most important driver of their attendance at markets, followed by financial incentives and exposure interventions. Review of staff records regarding activities offered at each market indicated high participant attendance of children’s activities and collection of educational materials by parents, reinforcing the evidence that participants highly valued the education and exposure interventions offered by HFHF. The lack of statistically significant associations between dietary change, attendance, and monetary incentives is likely due to the small sample size of exit surveys and minimal between person variation in dietary change.

## Limitations

A major limitation of this evaluation is the lack of a control group, which was not deemed feasible since waitlisted participants did not participate in surveys in adequate numbers. These findings may not be generalizable to the broader SNAP and WIC recipient population because of demographic, geographic and community factors that may specifically encourage farmers’ market attendance. Also, dietary intake was reported in “times per day” instead of “servings per day,” limiting comparison with several traditional reference studies.

Although the exit sample was statistically representative of the entire participant group, the approach of capturing participants at all markets on specific days required by staffing constraints allows the chance of some small amount of selection bias. The high self-reported consumption levels of F&V prior to program participation may have diminished dietary intake changes resulting from program participation. Participants also may have overestimated F&V intake and underestimated soda intake, in part, because of social approval bias.^17^ However, while research indicates that self-reporting may not accurately capture consumption, it is valid for ranking intake between participants and in paired samples via non-parametric methods.^18,19^

## Conclusion

Urban farmers’ market-based interventions combining exposure activities and small financial incentives can improve diet quality of low-income families. This study reinforces the existing evidence that even relatively small amounts of financial incentives that directly target F&V purchasing can be effective in helping low-income families to increase F&V intake. Importantly, however, HFHF also employed exposure and education interventions in addition to financial incentives, with participants reporting high engagement and use of the program to supplement existing WIC/SNAP F&V purchasing, instead of replacing it.

The potential of expanding the use of farmers’ markets as an intervention setting bears further examination. The social and cultural norms and values asserted in such an environment may play a reinforcing role that increases the efficacy of incentive programs and exposure interventions, warranting further research. Additional research into the tangible health effects of increased F&V consumption resulting from access and exposure improvements for target populations is warranted.

## Ethical approval

The study protocol was reviewed and exempted by the Harvard T.H. Chan Institutional Review Board.

## Competing interests

None to be declared.

## Acknowledgements

The 2013 HFHF Program itself was made possible with generous support from Blue Cross Blue Shield of Rhode Island, the van Beuren Charitable Foundation, the Fresh Sound Foundation, Seven Stars Bakery and CVS Caremark. The authors would like to recognize FFRI’s tireless staff members, the participating farmers, and families, without whom the study could not have been conducted.
